# Entropy Rules: Molecular Dynamics Simulations of Model Oligomers for Thermoresponsive Polymers

**DOI:** 10.3390/e22101187

**Published:** 2020-10-21

**Authors:** Alexander Kantardjiev, Petko M. Ivanov

**Affiliations:** Institute of Organic Chemistry with Centre of Phytochemistry, Bulgarian Academy of Sciences, ul. Acad. G. Bonchev, bloc 9, 1113 Sofia, Bulgaria; ivanov@bas.bg

**Keywords:** molecular dynamics, entropy calculation, thermoresponsive polymers, lower critical solution temperature, molecular dynamics, GROMACS—GPU calculations

## Abstract

We attempted to attain atomic-scale insights into the mechanism of the heat-induced phase transition of two thermoresponsive polymers containing amide groups, poly(*N*-isopropylacrylamide) (PNIPAM) and poly(2-isopropyl-2-oxazoline) (PIPOZ), and we succeeded in reproducing the existence of lower critical solution temperature (LCST). The simulation data are in accord with experimental findings. We found out that the entropy has an important contribution to the thermodynamics of the phase separation transition. Moreover, after decomposing further the entropy change to contributions from the solutes and from the solvent, it appeared out that the entropy of the solvent has the decisive share for the lowering of the free energy of the system when increasing the temperature above the LCST. Our conclusion is that the thermoresponsive behavior is driven by the entropy of the solvent. The water molecules structured around the functional groups of the polymer that are exposed to contact with the solvent in the extended conformation lower the enthalpy of the system, but at certain temperature the extended conformation of the polymer collapses as a result of dominating entropy gain from “released” water molecules. We stress also on the importance of using more than one reference molecule in the simulation box at the setup of the simulation.

## 1. Introduction

Most synthetic macromolecules become more soluble when heated. However, there are water-soluble polymers that separate from solution upon heating (inverse temperature-dependent solubility) above the phase transition temperature (lower critical solution temperature, LCST). Such polymers are referred to as thermoresponsive polymers. This phenomenon is explained to result from the balance between the enthalpy contribution from the energy stabilization due to hydrogen bonding of the polymer with the water molecules and the entropy gain of the system at higher temperature that outweighs the enthalpy preference at lower temperatures. Hydrogen bonding between the polymer and the water molecules lowers the free energy of dissolution. This effect becomes less important at higher temperature and, accordingly, entropy effects prevail [1].

The thermoresponsive polymers are characterized by the abrupt change in their properties in response to temperature variations [2,3]. Their solubility behavior is still a fundamental and challenging problem in polymer science, but remains elusive to describe theoretically and difficult to simulate via computational methods. A major challenge is to relate structural characteristics of the polymers to this phenomenon. Besides the fundamental theoretical issues, the thermoresponsive polymers are of significant technological importance for the design of stimuli-responsive (smart) materials with adaptive properties. Promising applications in the pharmaceutical industry present drug delivery, biomedical applications, e.g., tissue engineering and smart devices [4,5,6]. An important prerequisite for such applications is to fine-tune the temperature of the phase transition (e.g., aiming at the physiologically relevant human body temperature). Such control of the transition temperature point can be acquired by exploring copolymerization with other appropriate monomers. The LCST is the stringent formal thermodynamic characteristic of the thermoresponsive behavior. Below the LCST the components of the solution become miscible whatever the composition of the polymer solution. The effect at the molecular level is detected as a coil-to-globule transition of the conformation of the polymer.

**Figure 1:**
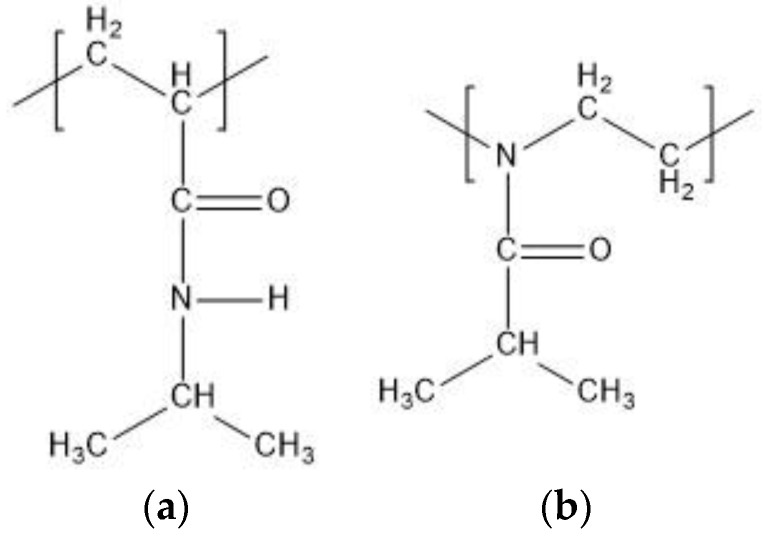
Schematic structures of (**a**) N-isopropylacrylamide (NIPAM) unit and (**b**) 2 isopropyl-2-oxazoline (IPOZ) unit.

Polymers containing amide groups constitute the largest group among the thermoresponsive polymers. The most examined thermoresponsive polymers are poly(N-isopropylacrylamide) (PNIPAM) and poly(2-isopropyl-2-oxazoline) (PIPOZ) (Figure 1). Both have an amide group in the side chain and the substantial difference between PNIPAM and PIPOZ is that the main chain of PNIPAM is nonpolar, whereas PIPOZ is with polar backbone. The amide groups of PNIPAM can be proton donors as well as proton acceptors, whereas the amide groups of PIPOZ can be only proton acceptors. We examine in this study by molecular dynamics simulations (MD) model oligomers of N-isopropylacrylamide (NIPAM) and 2-isopropyl-2-oxazoline (IPOZ).

The PNIPAM polymer is the paradigmic thermoresponsive molecule. The experimental research on the PNIPAM phase behavior in aqueous solution is abundant [1,2,3]. Still not so well examined is PIPOZ [7,8,9]. The PIPOZ polymer shows thermoresponsive behavior at around the same temperature range as PNIPAM. We focused our interest both on the thermodynamics and the mechanisms at the molecular level leading to the heat induced phase transition. The interest in the PIPOZ properties is additionally fueled by its advantages, relative to the PNIPAM polymer, in relation to practical applications, especially its biocompatibility.

Molecular dynamics simulations were also used to study the thermoresponsibility effect [10,11,12,13,14,15,16,17,18,19,20,21,22], but mostly in the case of PNIPAM and its copolymers. To the best of our knowledge only two published works report molecular dynamics simulations in the case of PIPOZ molecules [23,24]. The first one applied short atomistic MD runs of systems comprised of a single 8-mer PIPOZ molecule solvated by 500 water molecules and the focus is on the differences of the hydrogen bonding below and above the point of phase transition [23]. The other investigation applied MD simulation in vacuum in order to investigate the possible conformations of PIPOZ chains both in amorphous and crystalline states [24].

We apply in this study all-atom molecular dynamics simulations in an attempt to reproduce the phenomenology of the effect (i.e., temperature-induced collapse of the chain) for NIPAM and IPOZ oligomers in water solution aiming at getting structural and thermodynamic evidences for the cause of their heat induced phase transition behavior.

## 2. Methods

### 2.1. Molecular Mechanics Models of the Polymer Structures

The graphic antechamber [25,26] was used to build the molecular models for the PNIPAM and PIPOZ monomers. The generated structures were saved in the standard *mol2* format and the files were transferred to Gaussian input files [27] for geometry optimization and subsequent derivation of RESP (Restrained Electrostatic Potential) charges [28]. The Gaussian log file, containing the electrostatic potential distribution results, was used as an input file to the antechamber RESP functionality which performed the fit of the data and generated “*ac*” format file (short for antechamber) containing information about the derived atomic charges. In order to obtain monomer units with charge distribution corresponding to the monomer block within the polymer, we stripped out the charges of the linking methyl groups. A special file was generated manually that contains information about the linkage definitions, i.e., rules for removing those atoms of the monomer units which got stripped out upon polymerization. Special attention was devoted to the terminal residues of the polymer chain. The head and the tail residues cap the oligomer chain at the two ends. The same procedure as with the internal residues was followed to derive charges for the terminal residues. Two manually built files were used to define the rules for connectivity of head and tail residues. The AMBER *tleap* procedure was used to construct the oligomer systems. From this point on the GROMACS package [29,30,31,32] was used for the simulations. The AMBER topology was converted to the GROMACS formats by using the *AnteChamber PYthon Parser interfacE* [33]. The AMBER-03 [25] (a third generation force field, derived from AMBER99) all atom force field port for the GROMACS molecular dynamics program was used in the simulations.

### 2.2. Molecular Dynamics Setup Description

We conducted molecular dynamics simulations of fully atomistic models of NIPAM and IPOZ oligomers with chain lengths of 12 (12-mer), 24 (24-mer), and 30 (30-mer) monomer units in cubic simulation cells. The oligomers were solvated by filling the simulation box with water molecules using the *genbox* routine of the GROMACS suite. An explicit water model (TIP3P) was applied [34,35]. The systems were parameterized, assembled and equilibrated in several stages.

The total energies of the isolated oligomer chains were firstly minimized, followed by minimization of the energies of the whole systems, oligomers plus model water molecules. The energy of the system as a whole was minimized by means of the L-BFGS (limited-memory Broyden–Fletcher–Goldfarb–Shanno) algorithm. The optimized structure was used as the initial configuration for the simulations at two extreme temperature regimes—at 263.0 K and 333.0 K. The simulations were executed in the NPT (constant pressure and constant temperature) ensemble. Temperature was controlled by applying the Nose–Hoover thermostat with a coupling time 1.0 ps [36,37]. Periodic boundary conditions and minimum image convention were applied. The particle–particle particle–mesh method (P^3^M) [38] with a real space cutoff 12.0 Å was used for evaluating the electrostatic interactions. The choice of the P^3^M solver was motivated for its favorable *NlogN* computational complexity (where *N* is the number of all interaction sites in the system). The integrator used to propagate the molecular dynamics trajectories was the velocity Verlet algorithm. This choice was dictated by the availability of the efficiently parallelized version for GPU systems [39]. The integration time step was 2.0 fs. The lengths of the bonds involving hydrogen atoms were constrained by the LINCS procedure [40]. Two extreme cases for the simulation cell were explored for the 30-mer structures—a box with side length 100.0 Å and a larger box with a side of 300.0 Å.

Two initial geometries (an extended conformation and conformation optimized by conformational search [41]) of the NIPAM and IPOZ oligomers were used as startup conformations for the simulations of the 12-mers. For each of them five chains were randomly distributed in a cubic box (with 100.0 Å side). The multiple chain setup was deliberately designed in order to test computationally the agglomeration process concomitant with (or following) the thermoresponsive chain collapse. The specific molecular dynamics engine driving the simulation was the *mdrun* program of the GROMACS package version 4.6 [32]. The visualization engine throughout this work was the VMD program [42].

### 2.3. Data Extraction from Molecular Dynamics Trajectories

GROMACS routines were used to extract relevant structural data from the generated molecular dynamics trajectories. The dynamics of the conformational evolution of the oligomer chains were monitored by the variation with the simulation time of the end-to-end distance of the backbone chains and the radii of gyration (r_g_). The radius of gyration is a measure for the extent of a polymer chain and we applied it as a measure for the collapse of the polymer molecule into a globular state.

An analysis of the intermolecular (oligomer … water) hydrogen bonds was also carried out, with the purpose to correlate the hydrogen bonding patterns around the polymer molecules with the thermoresponsive behavior observed as a collapse of the polymer chain above the LCST.

### 2.4. Estimation of Entropy Contributions—Brief Theoretical Intermezzo

#### 2.4.1. Stringent Formal Definition—Sampling of the Complete Phase Space

Formally the entropy calculation seems straightforward—it is a logarithmic measure of the accessible volume in phase space):

(1) $$S=-k_{B}\langle \ln(\rho )\rangle$$

where ρ is the phase space density and *k_B_* is the Boltzmann constant, < > denotes ensemble average. However, in practice the direct calculation of the entropy value is infeasible. The difficulty stems from the entropy value dependence on the complete phase space of the system. Its evaluation requires an integral over the complete phase space which is computationally prohibitive.

(2) $$S=-k_{B}\int_{R}d^{3N}xd^{3N}p\rho (x,p)\ln[\rho (x,p)]$$

where *x*, *p* represent the coordinates and momenta of the particles in the ensemble.

#### 2.4.2. Access to Phase Space Density

An additional obstacle is the lack of knowledge of the phase space density. It is not directly accessible from molecular dynamics trajectories. Therefore, yet another major problem arises in the entropy calculation methods—finding a suitable approximation of the phase space density. Two approaches are relevant:

- **Phase space density estimation through energy and partition function**

The following relation gives access to the density through energy (which is accessible in the molecular dynamics simulation):

(3) $$\rho (x,p)=\frac{e^{-\beta H(x.p)}}{Z}$$

where *H* is the Hamiltonian and *Z* is the partition function of the system. However, again an obstacle arises—the cost for the evaluation of the partition function *Z*. Thus, we resorted to another approximation—the quasiharmonic approximation.

- **Quasiharmonic approximation—Gaussian analytical ansatz for density**

Alternatively, we can direct our thought at constructing an analytical ansatz of the density and fit it to the molecular dynamics’ trajectory. Karplus et al. [43] utilized the fact that the entropy can be decomposed into position dependent and moment dependent component. The momentum dependent component can be estimated analytically. Therefore, the bottleneck of this method is the computation of the coordinate dependent component. Again, direct estimation is impossible. However, fitting the density as an analytical ansatz to the molecular dynamics’ trajectory is a way around this. The density near local/global minimum on the free energy surface can be approximated by a Gaussian function. More specifically the generated molecular dynamics trajectories can be used to fit the following multivariate Gaussian function:

(4) $$\rho (x,p)=\frac{1}{{\left(2\pi \right)}^{3N/2}{\left|C\right|}^{\frac{1}{2}}}e^{-\frac{1}{2}{\left(x-x_{0}\right)}^{T}C^{-1}(x-x_{0})}$$

where *C* is the covariance matrix, **|***C***|** is the determinant of the covariance matrix. Actually, the fit parameters are the eigenvectors of the covariance matrix *C*—its 3*N* principal components. Then the entropy (based on the fitted density) can be obtained by the following formula:

(5) $$S=-\frac{3}{2}Nk_{B}+\frac{1}{2}k_{B}\ln\left[{\left(2\pi \right)}^{3N}\left|C\right|\right]$$

The covariance matrix of the atomic fluctuations has peculiar eigenvalue spectrum that might turn out to be problematic in realistic calculations. The problem comes from the contribution of the high-frequency motions. The solution comes from the analytic quantum mechanical treatment of the problematic degrees of freedom.

#### 2.4.3. Quantum Mechanical Considerations and Approximations

- **Quantum mechanical entropy of harmonic oscillator**

The solution of Schlitter et al. [44] excludes the low eigenvalues by application of quantum mechanical considerations for the entropy of the harmonic oscillator at room temperature

(6) $$\begin{array}{l} S=\frac{\kappa \alpha }{\exp(\alpha )-1}-\ln(1-\exp(-\alpha )),\\ \alpha =\frac{\hbar \omega }{kT} \end{array}$$

Quantum mechanical variance is related to the frequency *w* of the oscillator in the above expressions. On the other hand, data from molecular dynamics simulations can be used to estimate only the classical variance (no direct access to quantum mechanical variance).

- **Reducing the quantum formula to the application of the classical limit of the variance**

The classical limit of the coordinate variance can be substituted in the above expression of the entropy by application of the following relation [44]:

(7) $$\frac{1}{\alpha^{2}}=\frac{mkT}{\hbar^{2}}{\langle \Delta x^{2}\rangle }_{cl}$$

Then the entropy calculation boils down to the estimation of the classical variance (accessible from molecular dynamics simulations). The final expression for the entropy makes use of yet another heuristic approximation by Schlitter [44] and finally generalizing to the multidimensional case by summing over 3*N* degrees of freedom yields:

(8) $$S\approx \frac{1}{2}k_{B}\sum_{i=1}^{3N}\ln\left(1+\frac{k_{B}T}{\hbar^{2}}{\langle \Delta x_{i}^{2}\rangle }_{cl}\right)$$

where ${\langle \Delta x_{i}^{2}\rangle }_{cl}$ represent the classical variance of the ith degree of freedom.

In order to estimate the entropy contribution in the coil-to-globule transition, we applied a computational scheme based on these approaches—the quasiharmonic method of Karplus et al. [43] and the improvement based on Schlitter’s method [44]. The theoretical foundation and the computational bottleneck of the procedure is the evaluation of the covariance matrix of the Cartesian positional coordinates from the molecular dynamics trajectories. The entropy is calculated from the eigenvalues obtained after the diagonalization of the mass-weighted covariance matrix. Though not fully validated, the method had been already applied for entropy estimates in simulations of polymer molecules, e.g., to examine the entropy change upon protein folding [45].

## 3. Results and Discussion

Eight different setups for simulations were executed (Table 1). The discussion starts with examination of the structural variations with the simulation time of the 30-mer and the 24-mer NIPAM and IPOZ oligomer chains. Next, the results are presented for the multiple 12-mers (multiple chains) NIPAM and IPOZ molecules in an attempt to reproduce the temperature induced agglomeration of the multiple chains (in foil to the chain collapse). Molecular characteristics, hydrogen-bonding and polar contacts, were also determined and discussed. Special attention was devoted at the estimate of the entropy changes when passing from temperature far below (263.0 K) to temperature far above (333.0 K) the LCST point (for all cases, including 12-mer, 24-mer, and 30-mer NIPAM and IPOZ oligomers). The PNIPAM molecule undergoes a discontinuous hydration dehydration transition at around 305 K [1,2]. We consider the relatively wide span through the supposed transition temperature as necessary in order to obviate the mismatch between the temperature in the simulation settings and the real thermodynamic temperature.

**Figure 2:**
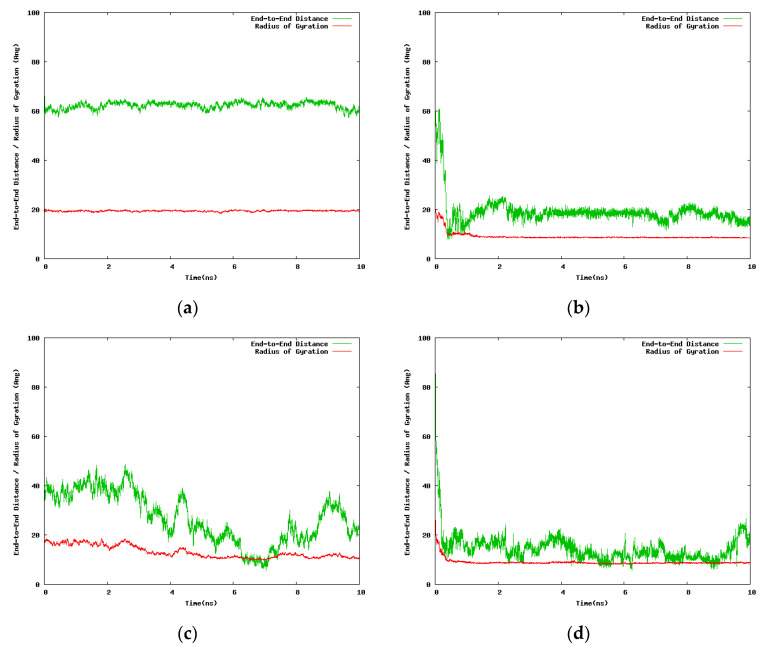
Variation with the simulation time of the end-to-end distance and the radius of gyration for the 30-mer NIPAM and IPOZ molecules, starting from an initially extended conformation in a cubic box with 300 Å side: (**a**) Simulation of NIPAM at 263.0 K; (**b**) simulation of NIPAM at 333.0 K; (**c**) simulation of IPOZ at 263.0 K; (**d**) simulation of IPOZ at 333.0 K.

**Figure 3:**
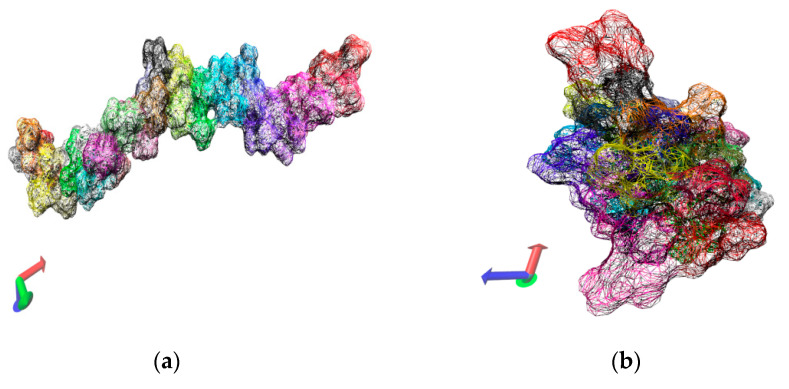

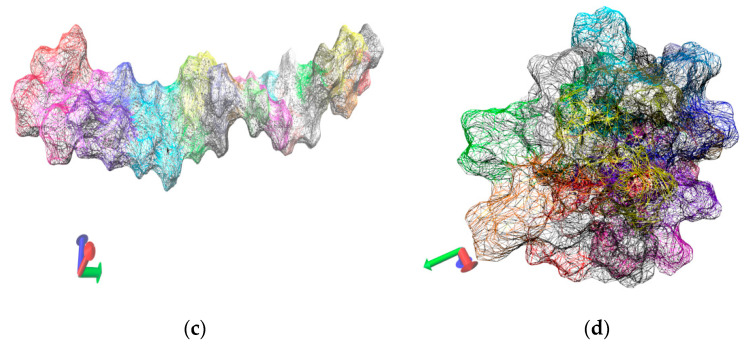
Variation of oligomer morphology with the simulation time for the 30-mer NIPAM (**a**,**b**) and IPOZ (**c**,**d**) molecules. The simulation time is 10.0 ns. The final snapshots extracted from the molecular dynamics trajectories are presented: (**a**) NIPAM at 263.0 K; (**b**) NIPAM at 333.0 K; (**c**) IPOZ at 263.0 K; (**d**) IPOZ at 333.0 K.

Two alternative initial setups were considered for the 30-mers with the purpose to assess the dependence of the results on the size of the solvent box, 100.0 Å and 300.0 Å side lengths, respectively. Due to computational limitations we were in a position to run only short simulations (10.0 ns) for the case of the large solvation box (300.0 Å). Although we generated additional simulation data for intermediate points along the temperature range, we report here only the results for the two extreme temperatures (263.0 K and 333.0 K). Figure 2 contains data extracted from the 10.0 ns simulation of single 30-mer NIPAM and IPOZ molecules starting from an initially extended conformation. There is clear tendency for collapse of the NIPAM oligomer chain at the higher temperature as the molecule adopts supposedly a globular state, whereas the variations of the radius of gyration and the end-to-end distance at the low temperature are in support for the persistence of the extended form (Figure 2a,b). The data for the 30-mer IPOZ molecule unequivocally demonstrate reproducibility of the experimentally observed LCST effect for PIPOZ solutions (Figure 2c,d). To the best of our knowledge, this is the first reported fully atomistic molecular dynamics reproduction (using adequate solvation and spatial scale) of the experimental observations for PIPOZ systems. Though relatively short, the simulation time is enough in order to show up the LCST effect. In order to get further clear picture at the molecular level, we illustrate with snapshots from the simulation trajectories the computed molecular structures in the final stages of the simulations for the two extreme temperature points (Figure 3). Both the NIPAM and the IPOZ oligomer chains show extended, though not strictly “coiled” conformations at the low temperature regime and even simple visual inspection at the higher temperature gives evidence for adopting globular form. Obviously, the globular conformations suggest dehydration of the polymer molecules due to diminishing of their contact surface with the surrounding solvent molecules.

**Figure 4:**
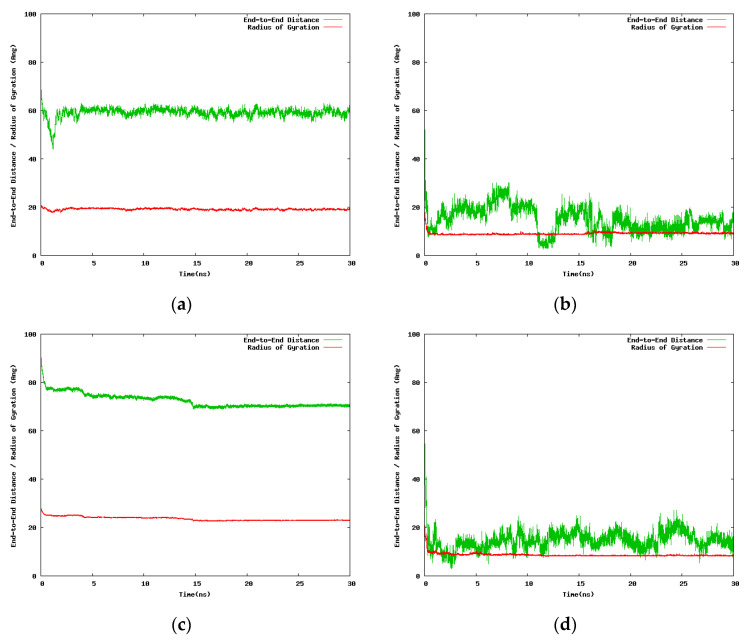
Variation with the simulation time of the end-to-end distance and the radius of gyration for the 30-mer NIPAM and IPOZ molecules, starting from an initially extended conformation in a cubic box with 100 Å side: (**a**) Simulation of NIPAM at 263.0 K; (**b**) simulation of NIPAM at 333.0 K; (**c**) simulation of IPOZ at 263.0 K; (**d**) simulation of IPOZ at 333.0 K.

The next set of NIPAM/IPOZ 30-mer simulations uses smaller cubic box with side 100.0 Å but the time scale was extended up to 30.0 ns. A caveat with using small simulation box is the problem with the application of periodic boundary conditions. In the case of a small box size the NIPAM/IPOZ oligomer molecules might interact with the chain image in the adjacent periodic cell. This artificial interaction might produce nonphysical effects—periodic images are identical physical objects and the produced effect is actually self-interaction. We took care to check for artificial interactions between neighboring periodic images in order to avoid nonphysical effects in the simulation results. Figure 4 illustrates the time dependence of the large scale structural changes for the NIPAM and IPOZ 30-mer oligomers occurring at the two extreme temperatures.

Special care had to be taken in order to ascertain that no direct interactions between the oligomer molecules and their periodic images could take place. For this purpose we regularly checked the minimal distance between periodic images by using the *mindist* routine of the GROMACS suite. However, the indirect effects from the interactions of the periodic images are quite difficult to monitor and list out. These hard to follow interactions are indirect in the sense of being mediated through the structure of the surrounding solvent molecules, namely the ordering of water molecules at the nanometer range. Beyond these considerations the results are unequivocally clear that at high temperatures the 30-mer IPOZ chain experiences collapse as monitored by the abrupt change in the radius of gyration/end-to-end distance, which is in support for the PIPOZ thermoresponsive behavior. Data displayed in Figure S1 (in Supporting Information) supports the above conclusions for the 24-mer NIPAM and IPOZ cases.

We used alternative initial setups for the 12-mer case corresponding to extended and optimized conformations of the oligomer molecules. In addition, the simulation box contains five oligomers, thus we attempted to reproduce the effect of agglomeration (concomitant with the chain collapse). Figure S2 (Supporting Information) presents data for NIPAM and IPOZ starting from an initially extended conformation. A tendency is evident for polymers to collapse at high temperatures as the molecule strives to adopt a globular state (Figure S2b). Although slight, the shrinking of the structure is clearly present above LCST. The same data series for the IPOZ oligomers (Figure S2d) at the high temperature point also indicates a slight tendency for getting smaller average extent of the molecule as a consequence of the adoption of a globular structure. At the low temperature point (Figure S2a,c) the MD trajectories end with structures which preserve some of the spatial extent of the oligomer molecule which is a sign that temperatures below LCST favor the coil hydrated state. Thus, the simulations for the 12-mer IPAM and IPOZ molecules also provide evidences for thermoresponsive type behavior.

**Figure 5:**
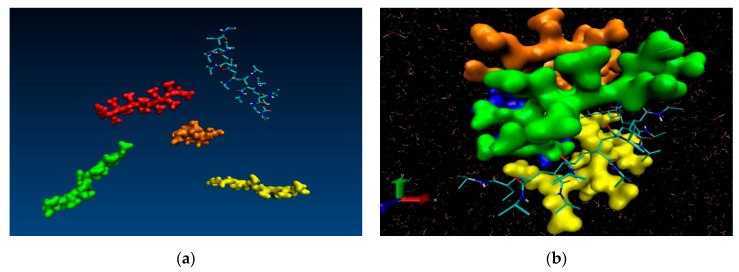
Visualization of the 12-mer NIPAM morphology in solution. The simulation time is 12.0 ns. Snapshots of the first and the last frames are shown: (**a**) The five NIPAM 12-mers with extended initial conformations; (**b**) a snapshot at the end of the simulation carried out at high temperature point.

Besides the process of polymer chain collapse at the single molecule level, an additional phenomenon manifests itself in the molecular dynamics’ simulations of multiple chains, namely agglomeration of the collapsed chains. The collapse of the polymer chain is a prerequisite for the aggregation and the precipitation of the solution thus the two processes can be considered concomitant. At phenomenological level it can be observed as shrinkage of the polymer microgel. Our multiple chain simulations are illustrative in this respect and reproduce faithfully the experimentally observed agglomeration. Figure 5 contains snapshots extracted from the simulation trajectory of NIPAM oligomers using an extended starting conformation, leading finally to chain collapse at 333.0 K. The visual inspection in a molecular viewer (VMD) supports the phenomenon of low critical solution point phase transition. A comparison of the first snapshot (dispersed chains in solution) and the last snapshot of the trajectory (i.e., at 0.0 ns and 12.0 ns, respectively) witnesses the collapse of the oligomer chains and their consequent aggregation which experimentally is registered as insolubility—formation of aggregates above the transition temperature point. Analogous results (computational reproduction of agglomeration) were reported for yet another *N*-substituted acrylamide-based polymer—poly(*N*-*n*-propylacrylamide) (PnnPAm) which is a structural isomer of the poly(*N*-isopropylacrylamide) [46].

The dependence of the results on the starting conformations was also examined. Figure S3 displays the results for the 12-mer NIPAM and IPOZ molecules, starting from an initially optimized conformation [41]. There is slight tendency for both oligomers to unfold at low temperature conditions. This result is an interesting computational evidence for the thermoresponsive effect in the reverse direction—the case with initial pre-collapsed chain conformation which strives to adopt extended state when subjected to low temperature. Previous attempts to demonstrate the effect for initially collapsed NIPAM oligomer chain failed to observe such behavior, even at very long duration of the simulation—1.0 μs [47]. A possible reason could be hidden behind their MD setup with a single chain NIPAM oligomer [47] against the multiple chains’ simulations in our case. The low temperature simulation of the IPOZ oligomer (with optimized starting conformation) also shows signs for computational reproduction of the thermoresponsive effect: As expected the simulations at the low temperature regime lead to increase of the end-to-end distance (Figure S3d). However, obtaining extended final coil state in a hydrated (a swollen coil) conformation is beyond reach in our simulation setup since the effect could be fully revealed in computations with much longer time scales.

Next, we looked for details about the solvent structure around the hydrophobic and the hydrophilic moieties of the oligomers. More specifically, in order to elicit the factors at molecular level that govern the thermoresponsive behavior we examined the modes of solvent–polymer interactions in terms of hydrogen-bonding and overall polar contacts. Supposedly the major role in the process has to be ascribed to the so-called “bound water”. One can differentiate two types of bound water molecules: Hydrogen bonded water molecules with the –C=O or –N–H groups of the polymer molecule and interactions that can take place with the participation of the hydrophobic groups—methyl groups or the main-chain skeleton. As far as hydrophobic ordering is concerned, we tackle the issue of solvation patterns and reorganizations around hydrophobic moieties in terms of entropy contribution. Actually, the organization of the proximal solvent molecules is reflected in the entropy calculations (vide infra). The detailed local structure of the water clathrates around the polymer molecule is beyond the scope of our examination.

We consider atoms X–H … Y (X, Y–N or O) to constitute a hydrogen bond if they fulfill the following requirements: The donor–acceptor distance X … Y is less than 3.6 Å, the hydrogen–acceptor distance is less than 2.45 Å, and the angle between the donor–hydrogen vector and the donor–acceptor vector is less than 30.0°. The water–polymer hydrogen bonds are of two types: The polymer molecule provides the donor group and the water oxygen atoms are acceptors in hydrogen bonding, or the polymer molecule participates with acceptor groups and the water molecules are the donors in the hydrogen bond pair. We estimated also the number of pairs of oligomer–solvent polar atom contacts.

**Figure 6:**
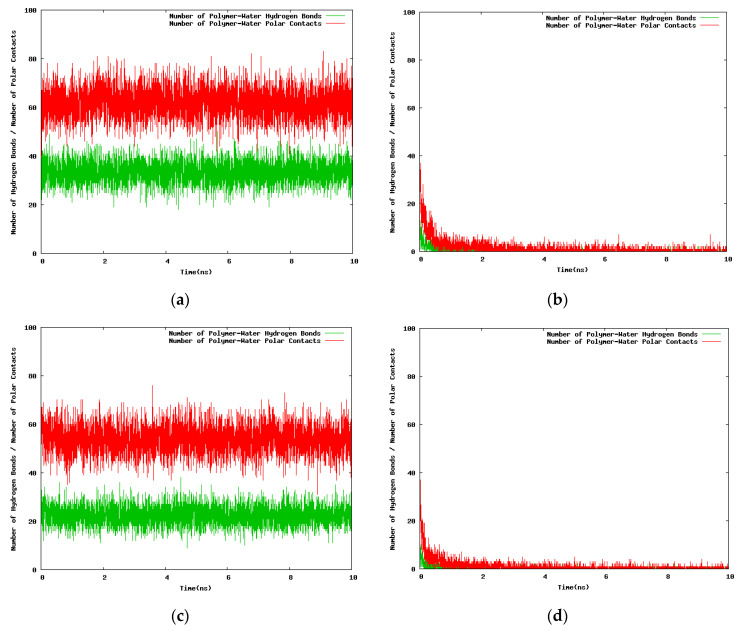
Variation with the simulation time of the number of hydrogen bonds and the contacts between polar atoms of the 30-mer NIPAM/IPOZ molecules and the solvent molecules, starting from initially extended conformations in a cubic box with 300 Å side: (**a**) Simulation of NIPAM at 263.0 K; (**b**) simulation of NIPAM at 333.0 K; (**c**) simulation of IPOZ at 263.0 K; (**d**) simulation of IPOZ at 333.0 K.

**Figure 7:**
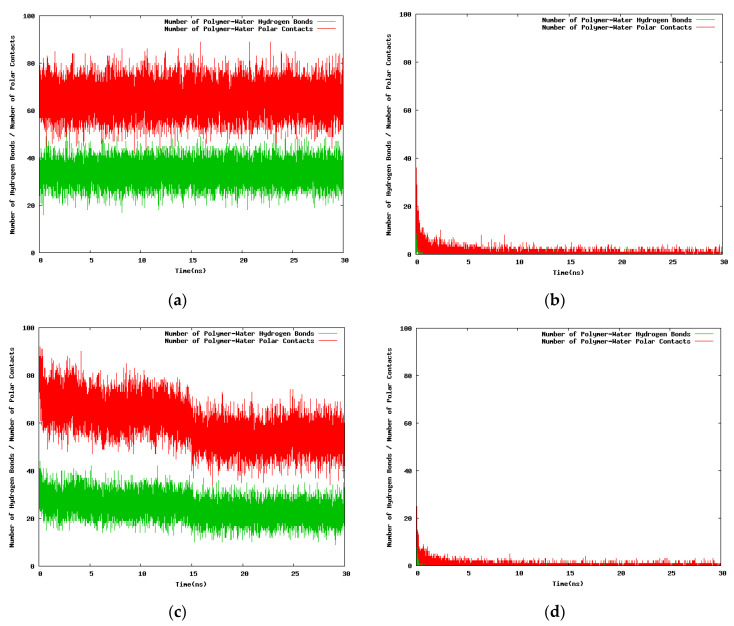
Variation with the simulation time of the number of hydrogen bonds and the contacts between polar atoms of the 30-mer NIPAM/IPOZ molecules and the solvent molecules, starting from initially extended conformations in a cubic box with 100 Å side: (**a**) Simulation of NIPAM at 263.0 K; (**b**) simulation of NIPAM at 333.0 K; (**c**) simulation of IPOZ at 263.0 K; (**d**) simulation of IPOZ at 333.0 K.

The results for the variation with the simulation time of the hydrogen bond number and the polar contacts for the 30-mer, 24-mer, and 12-mer NIPAM/IPOZ oligomer molecules are presented in Figure 6 and Figure 7 (and in Figures S4 and S5). The data is consistent with the thermoresponsive effect. The number of hydrogen bonds remains constant at the low temperature (263.0 K) (Figure 6a,c and Figure 7a,c and Figures S4a,c and S5a,c), whereas it diminishes sharply with the simulation time at high temperature (333.0 K) reflecting the folding process of the oligomer chains with concomitant breaking of water-oligomer hydrogen bonds (Figure 6b,d and Figure 7b,d and Figures S4b,d and S5b,d). The time dependence of the polar contacts follows closely the hydrogen bond number curve in all cases.

**Figure 8:**
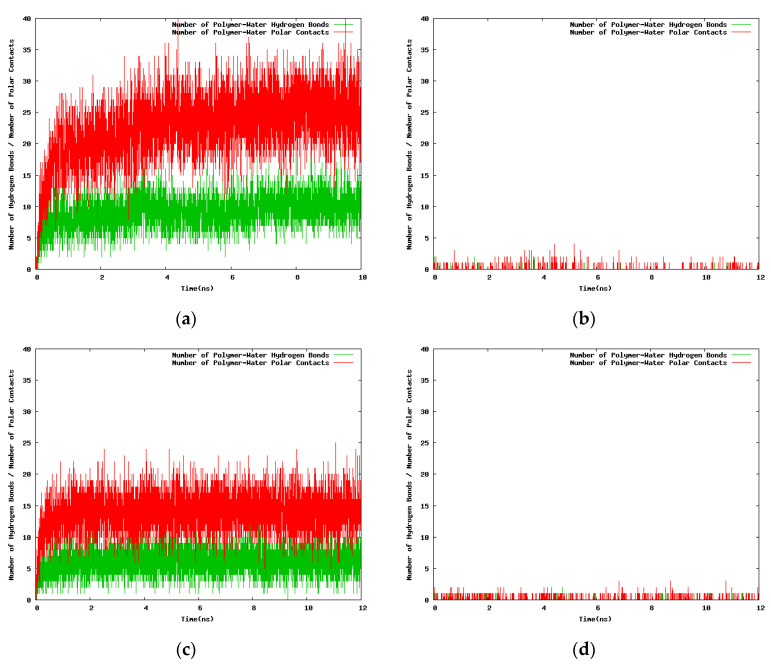
Variation with the simulation time of the number of hydrogen bonds and the contacts between polar atoms of the 12-mer NIPAM/IPOZ molecules and the solvent molecules, starting from initially optimized conformations in a cubic box with 100 Å side: (**a**) Simulation of NIPAM at 263.0 K; (**b**) simulation of NIPAM at 333.0 K; (**c**) simulation of IPOZ at 263.0 K; (**d**) simulation of IPOZ at 333.0 K.

With the next set of time series (Figure 8) we seek to find differences in the time dependent hydrogen bonding patterns for the NIPAM and the IPOZ oligomers when the starting conformation of the oligomer molecule is optimized from conformational search [41]. At high temperature (333.0 K) the number of both PNIPAM-water (Figure 8b) and PIPOZ-water (Figure 8d) hydrogen bonds remains constant (very low) with the simulation time, as expected for the folded state of a thermoresponsive polymer at high temperature. The rapid increase of the oligomer–water hydrogen bonds number at low temperature in the first few nanoseconds reflects the process of unfolding for both PNIPAM (Figure 8a) and PIPOZ (Figure 8c). Evidently, the effects are even more strongly pronounced when using initially optimized geometries.

For the sake of delving deeper into the thermodynamic factors governing the phase transition, we focused on estimating the entropy component of the free energy as a possible reason for the thermoresponsive behavior. Such a scenario consistently explains the reverse temperature effect: If the change of entropy is positive (increase of entropy), then with increasing the temperature the free energy of the system will change favorably (diminishes due to the term *–T*Δ*S*). Moreover, one can decompose the entropy effect into separate contributions that provides means to gain further insight into the mechanism of the thermoresponsive phase transition. The entropy change can be decomposed either in terms of the separate system components (e.g., solute vs. solvent) or estimated as contributions corresponding to the classification of the degrees of freedom of the system as translational, rotational and vibrational. What we attempted was to determine the entropy differences of the oligomer chains at two temperatures, one of them below and the other above the LCST, and to compare it with the entropy change related to the water solvent shell. Our estimates are based on the approach of Schlitter [43,44]. The covariance matrix of the positional fluctuations of the atoms (the *g_covar* routine of the GROMACS suite was used) was determined for the purpose, i.e., the configurational entropy was extracted from the fluctuations of the 3N-6 internal degrees of freedom of the oligomer. The elements of the covariance matrix are time averages over the configurations along the molecular dynamics’ trajectory. Then the Schlitter’s formula was applied by using the eigenvalues/eigenvectors of the covariance matrix [44]. The estimation of the entropy changes of the solvent also poses specific problems, originating from the diffusive motion of the water molecules which leads to enlargement of the configurational space available to the solvent. Here again we evaluated the Cartesian-coordinate covariance matrix.

**Figure 9:**
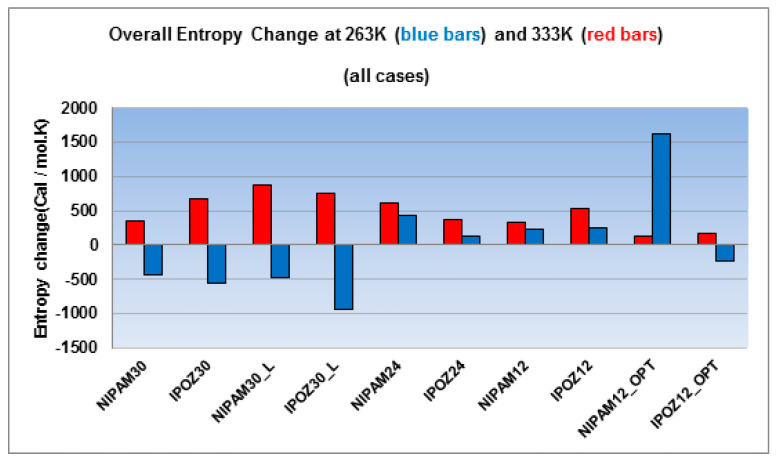
The overall entropy changes for the 30-mer, 24-mer and 12-mer NIPAM and IPOZ systems (NIPAM30_L and IPOZ30_L refer to simulations with large box size (300 Å side); NIPAM12_OPT and IPOZ12_OPT refer to simulations with initially optimized forms). The figure displays the results from simulations at two extreme temperature points—at 263.0 K (blue bars) and at 333.0 K (red bars).

Figure 9 displays the computed total entropy changes (including both, the oligomer and the solvent contributions) at 263.0 K and 333.0 K. For all 30-mer cases the calculated entropy change is negative at the low temperature and it is positive for the simulations at the high temperature regime. The data unequivocally supports the “entropy driven” hypothesis for the thermoresponsive effect. On the other hand, the case of 24-mers and 12-mers is characterized with positive total entropy changes at high and at low temperatures. However, the entropy change at 263.0 K is smaller in comparison to the 333.0 K and thus entropy is still favorable for the folding events at high temperatures in this case. This difference is even more pronounced in terms of the entropy contribution to the free energy change, since the entropy term is given higher weight with heightening of the temperature (due to the *–T*Δ*S* term). Thus, even close values of the entropy variation may lead to significant differences in the free energy change of the process. Therefore, the sets of 24-mer and 12-mer data is also in support for the decisive role of the entropy change as the key thermodynamic factor responsible for the collapse of the polymer at high temperatures. It is honest to mention that this data set contains a peculiar point—NIPAM12_OPT large entropy change which cannot be logically explained.

We have to keep in mind the approximations underlying the quasiharmonic approach, namely not accounting for anharmonicities of the vibrations and the correlations of the probability distributions. A necessary condition for convergence to realistic entropy values is the adequate phase space sampling which could be controlled but may require long simulation times. Besides, adequate sampling is problematic for rugged and frustrated energy surfaces—it is tricky to visit all relevant energy wells and estimate their relative populations. Therefore, an important limiting factor appears to be a system specific property—the properties of the energy surface. It is supposed to be approximated by quadratic function of the internal coordinates. This condition is severely critical for the solvent dynamics. If the water molecules’ dynamics do not deviate from harmonic approximation (which validates the analysis of the covariance matrix) we can give credit to the reported computational results and consequent conclusions. Unfortunately, the literature is scant for data on the numerical accuracy of the approach. One possible avenue for future improvements of the accuracy of the results is the inclusion of the cubic anharmonic terms [48]. Assuming that our systems meet these conditions, we can proceed with this method to dissect further the entropy contributions.

**Figure 10:**
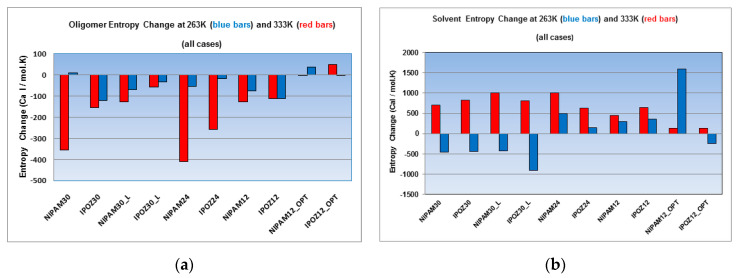
Decomposition of the overall entropy change into (**a**) oligomer contribution and (**b**) solvent contribution. The figure displays the results from simulations at two extreme temperature points—at 263.0 K (blue bars) and at 333.0 K (red bars). Entropy calculations were carried out for the 30-mer, 24-mer and 12-mer NIPAM and IPOZ systems (NIPAM30_L and IPOZ30_L refer to simulations with large box size (300 Å side); NIPAM12_OPT and IPOZ12_OPT refer to simulations with initially optimized forms).

Next, we estimated the variation of the entropy in a decomposed mode, namely for the oligomer component (Figure 10a) versus the solvent component (Figure 10b) for the two extreme temperature points—263.0 K vs. 333.0 K. Both the NIPAM and the IPOZ oligomers show loss of configurational entropy of the backbone chain upon its collapse at high temperature for all cases, except for the 12-mer IPOZ with optimized initial conformation. However, the concurrent entropy change of the solvent increases in all cases (Figure 10b) and compensates for the entropy loss of the oligomer—in total the entropy change is positive as shown in Figure 9. The backbone chain entropy changes at low temperature are negative for most cases. A notable exception is the 12-mer IPAM oligomer with optimized initial conformation. However, this is not surprising, because the thermoresponsive oligomer is supposed to strive to unfold at low temperature and increase its absolute entropy thus making the entropy change for the process positive. The solvent entropy change for the low temperature series is markedly convenient for explaining the thermoresponsive effect in the 30-mer case, i.e., the negative entropy change contribution which overcomes considerably the positive entropy change of the oligomers, makes temperature induced folding unfavorable. The case of the solvent entropy values for the 24-mers and 12-mers at low temperature can be discussed in comparison with the high temperature point results and explained as done above for the total entropy changes in order to apply the entropy calculations results for explanation of the thermoresponsive effect.

On the other hand, a possible explanation at the molecular level might be given in terms of the solvent-solvent interactions in the presence of exposed functional groups of the polymer in the extended conformation, and compared with the inaccessibility of these functional groups by the solvent in the collapsed globular state. For the extended form of the polymer, at temperatures below the LCST, the solvent molecules organize themselves in ordered structures of water clusters around the exposed polymer functional groups linked via hydrogen-bonded networks with the polymer. It had been shown that the solvent molecules of the first solvation shell are involved in a hydrogen bond network characterized by hydrogen bonding bridges between neighboring isopropylamide groups in a way that favors an extended conformation of the PNIPAM molecule below LCST [49]. Such ordered structures contribute for the lowering of the overall entropy of the system by restricting the degrees of freedom of the system. This arrangement of water molecules is no longer stable above the transition temperature point. The additional degrees of freedom upon release of solvent molecules raise the entropy significantly. With increasing the temperature, increases the weight of the entropy contribution to the overall free energy of mixing, whereas when the free energy change is positive then the observed effect is phase separation. According to our results, the entropy of the solvent is the decisive factor for the thermoresponsive behavior of the PNIPAM/water and the PIPOZ/water binary solutions.

## 4. Conclusions

We attempted to elucidate the mechanism of the heat-induced phase separation in two thermoresponsive polymers containing amide groups, PNIPAM and PIPOZ, and we succeeded in reproducing the existence of transition temperature point. We found out that the entropy has an important contribution to the thermodynamics of the phase separation transition. Moreover, we revealed that the entropy of the solvent has the decisive share for the lowering of the free energy of the system when increasing the temperature above the LCST. The water molecules structured around the functional groups of the polymer that are exposed to contact with the solvent in the extended conformation lower the enthalpy of the system, but at certain temperature the extended conformation of the polymer collapses as a result of dominating entropy gain from “released” water molecules. In addition to further longer simulations, one could expect that upon increasing the number of polymer chains in the simulation box and the size of the oligomer molecules the effect of the temperature change on the polymer conformation and consequent aggregation of the solute molecules will be more clearly pronounced.

## Figures and Tables

**Table 1:** The simulation setups executed depending on the number of monomeric units, the number of oligomer molecules in a unit box and the size of the cubic box with model water molecules.

	PNIPAM				PIPOZ			
Number of oligomer molecules in the unit box	1		2	5	1		2	5
Number of monomeric units	30		24	12	30		24	12
Size of the cubic box with water molecules [Å]	100.0	300.0	100.0	100.0	100.0	300.0	100.0	100.0
Number of water molecules	11,748	294,902	11,019	10,980	11,733	294,887	11,010	10,945
Duration of the simulation [ns]	30.0	10.0	12.0	12.0	30.0	10.0	12.0	12.0

## Supplementary Materials

The following are available online at https://www.mdpi.com/1099-4300/22/10/1187/s1, Figures presenting the variation with the simulation time of the end-to-end distance, the radius of gyration and the polymer–water hydrogen bonds. A section describing AMBER03 force field with explicit functional form.
