# Supplementary material for: Entropy Rules: Molecular Dynamics Simulations of Model Oligomers for Thermoresponsive Polymers
Source: Entropy (Basel). 2020 Oct 21;22(10):1187. doi: 10.3390/e22101187 (PMC7597358; doi:10.3390/e22101187)
Supplement: Supplementary file 1 [file entropy-22-01187-s001.pdf]

SUPPORTING INFORMATION

Entropy Rules: Molecular Dynamics Simulations of Model Oligomers for Thermoresponsive Polymers

Alexander Kantardjiev,* Petko M. Ivanov

Institute of Organic Chemistry with Centre of Phytochemistry, Bulgarian Academy of Sciences, ul. Acad. G. Bonchev, bloc 9, 1113 Sofia, Bulgaria

E-mail: alexkant@orgchm.bas.bg

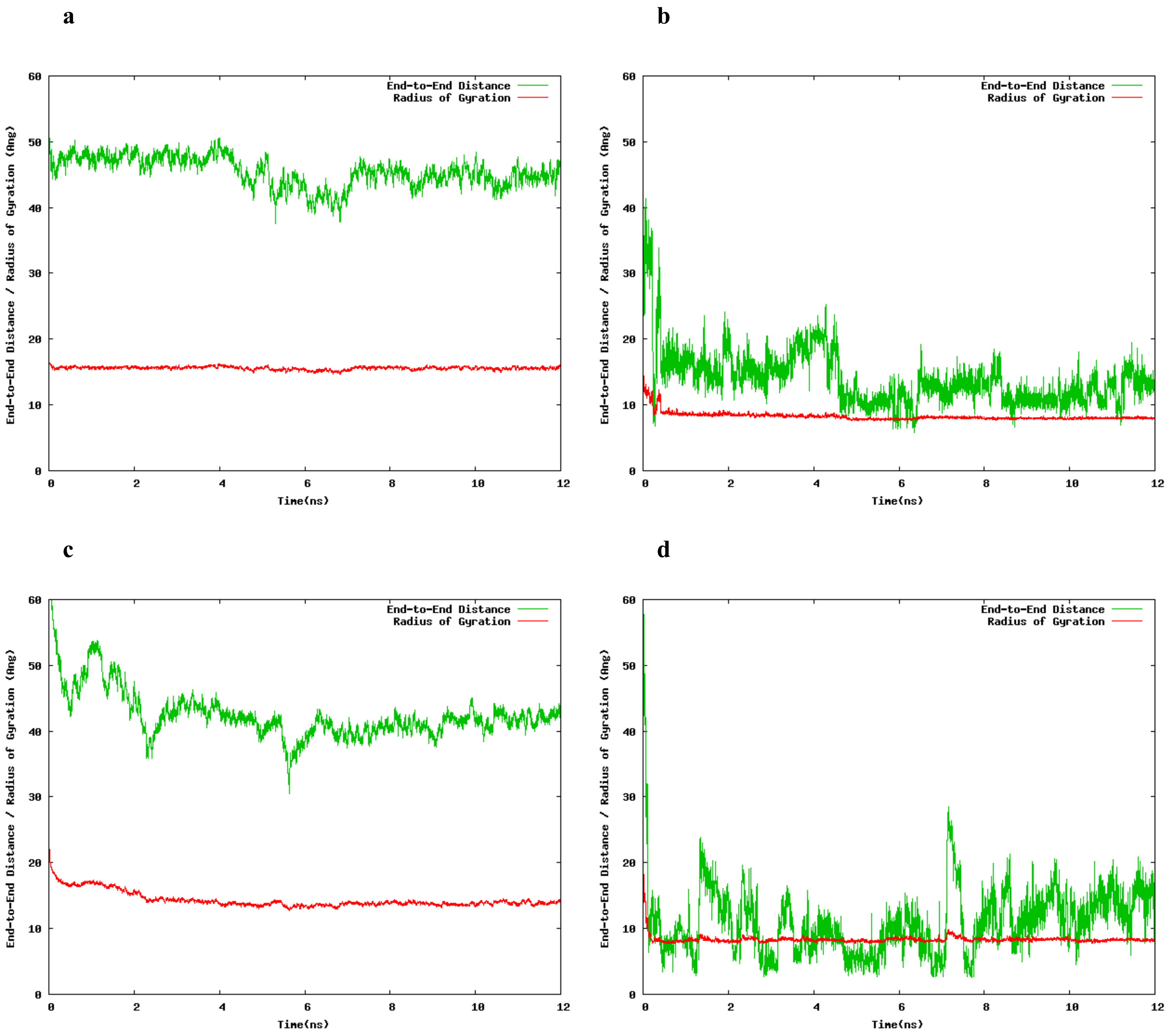

Figure S1. Variation with the simulation time of the end-to-end distance and the radius of gyration for the 24-mer NIPAM and IPOZ molecules, starting from an initially extended conformation in a cubic box with 100 Å side: (a) simulation of NIPAM at 263.0 K; (b**)** simulation of NIPAM at 333.0 K; (c) simulation of IPOZ at 263.0 K; (d) simulation of IPOZ at 333.0 K.

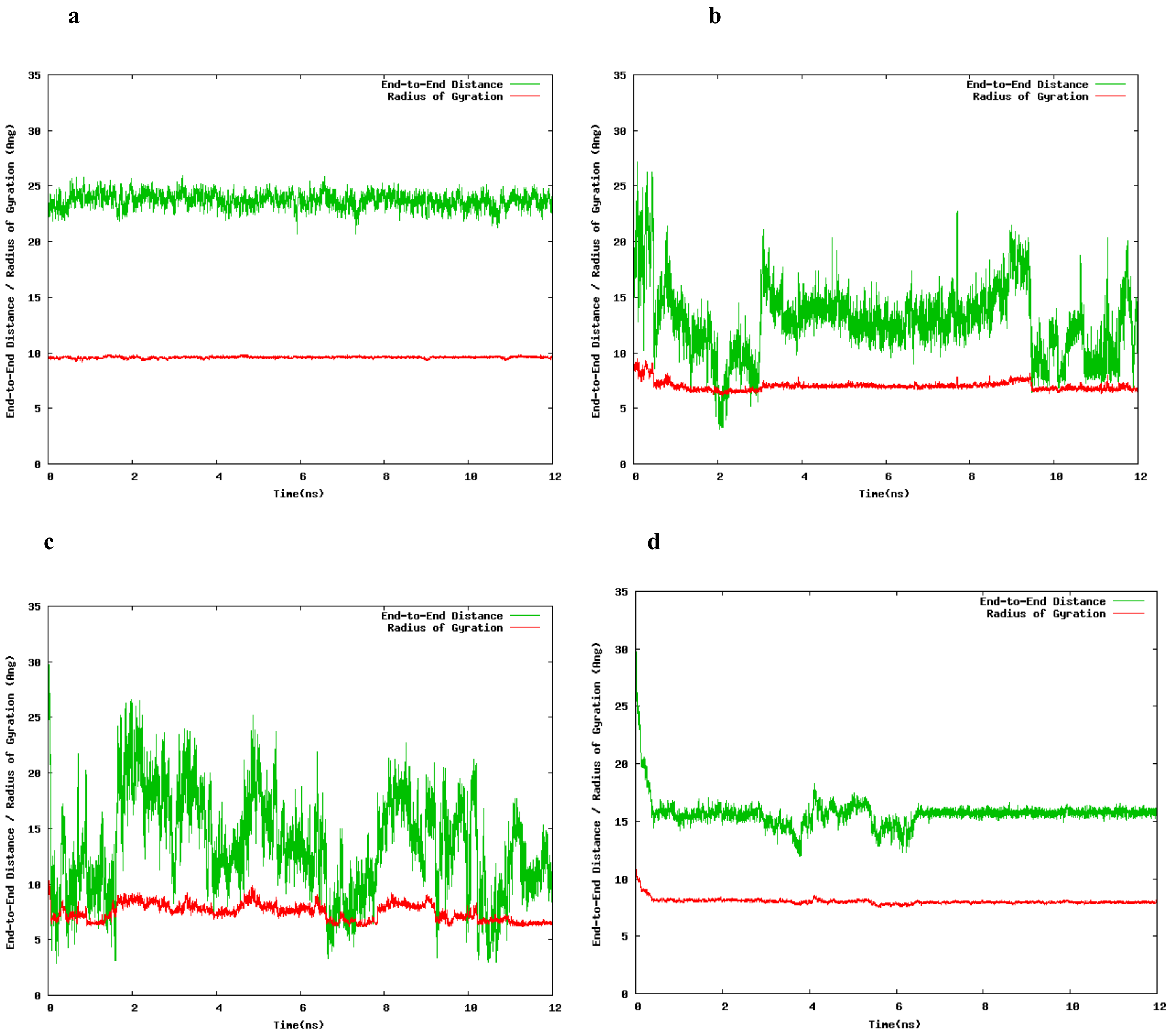

Figure S2. Variation with the simulation time of the end-to-end distance and the radius of gyration for the 12-mer NIPAM and IPOZ molecules, starting from an initially extended conformation in a cubic box with 100 Å side (the data is averaged over the five oligomers in the simulation box): (a) simulation of NIPAM at 263.0 K; (b) simulation of NIPAM at 333.0 K; (c) simulation of IPOZ at 263.0 K; (d) simulation of IPOZ at 333.0 K.

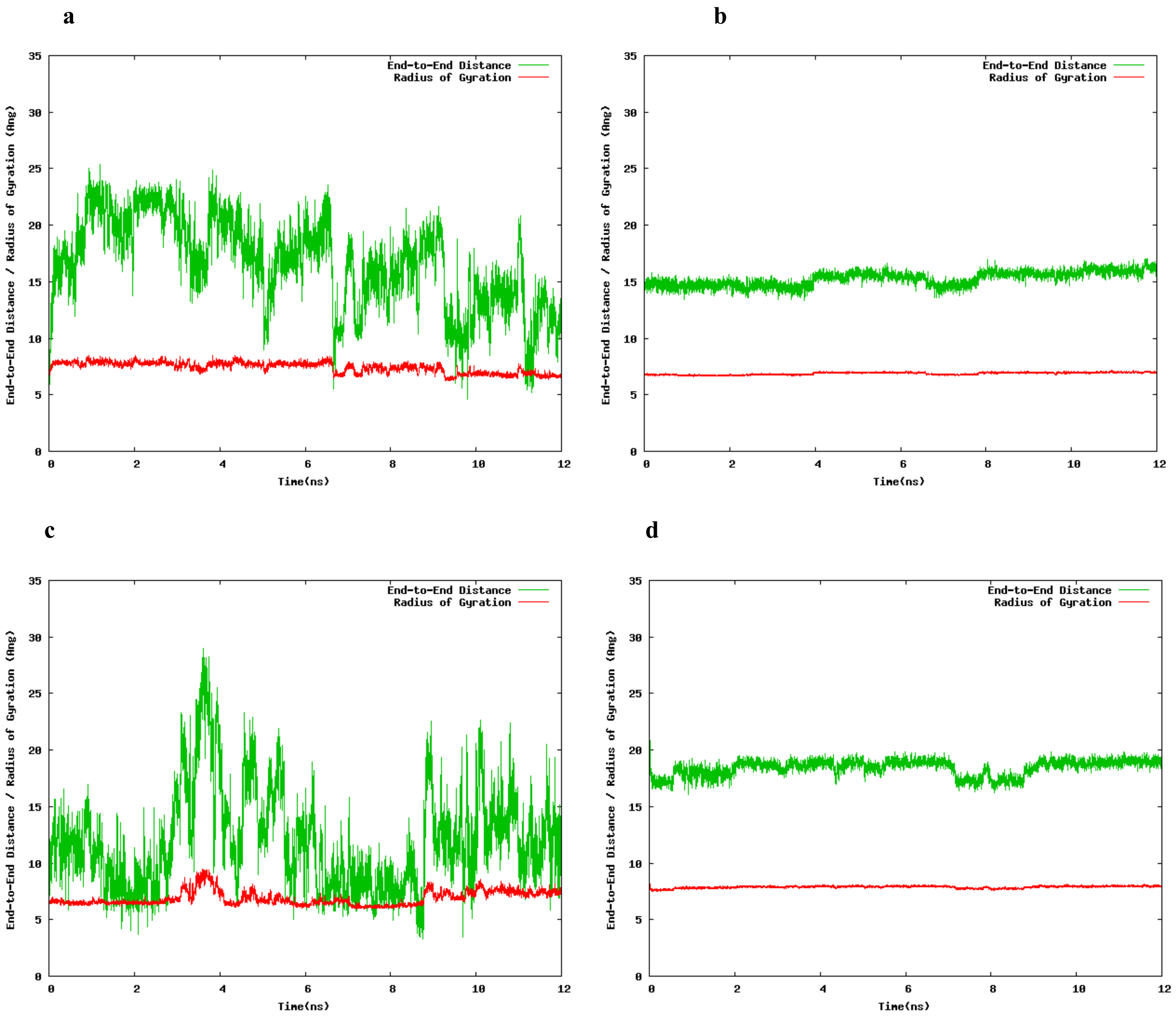

Figure S3. Variation with the simulation time of the end-to-end distance and the radius of gyration for the 12-mer NIPAM and IPOZ molecules, starting from an initially optimized conformation in a cubic box with 100 Å side (the data is averaged over the five oligomers in the simulation box): (a) simulation of NIPAM at 333.0 K; (b) simulation of NIPAM at 263.0 K; (c) simulation of IPOZ at 333.0 K; (d) simulation of IPOZ at 263.0 K.

a

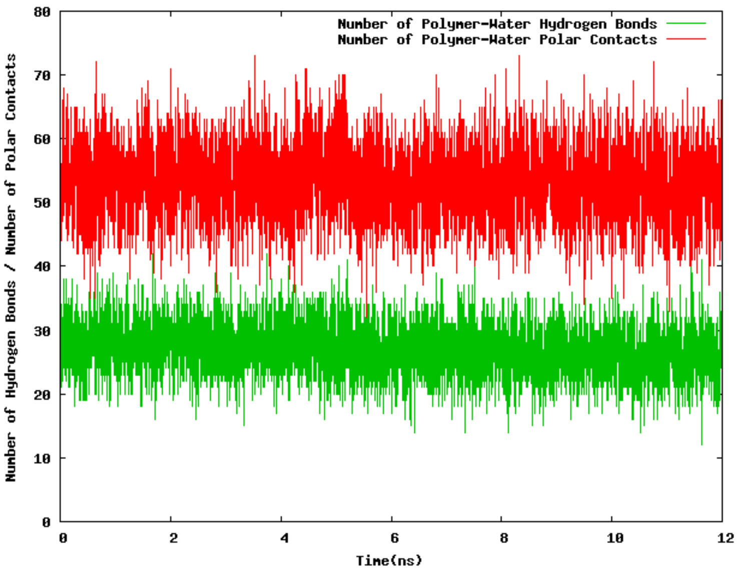

b

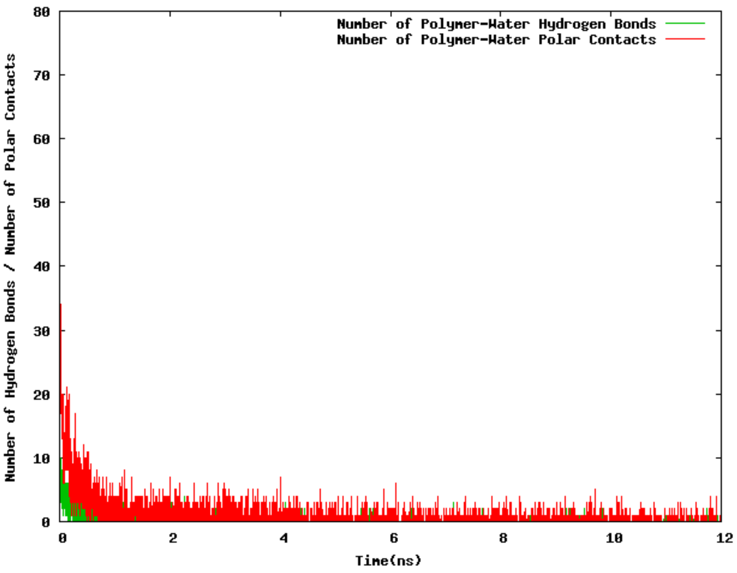

c

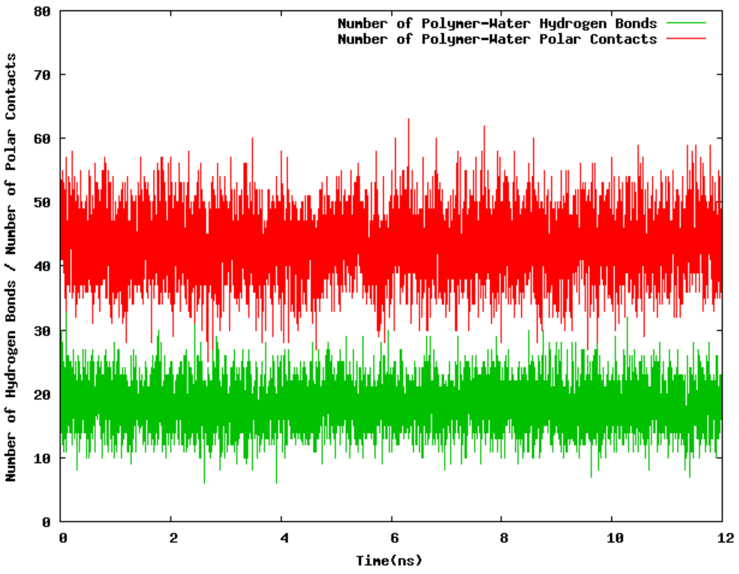

d

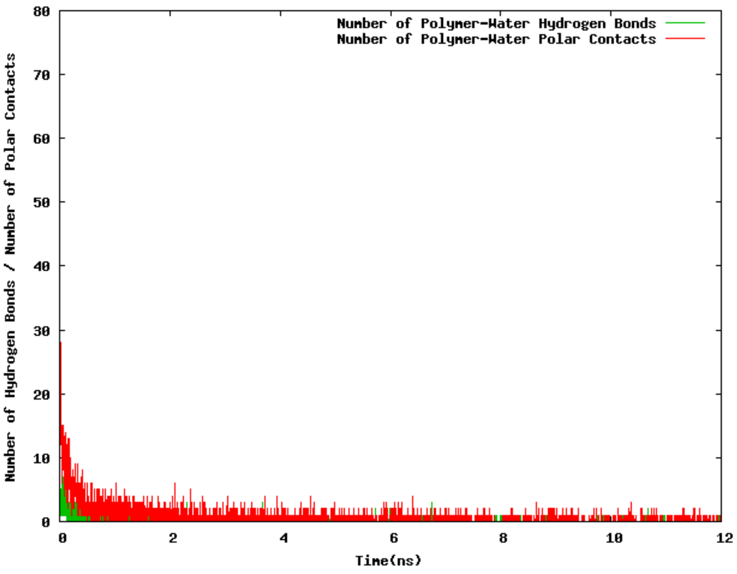

Figure S4. Variation with the simulation time of the number of hydrogen bonds and the contacts between polar atoms of the 24-mer NIPAM / IPOZ molecules and the solvent molecules, starting from initially extended conformations in a cubic box with 100 Å side: (a) simulation of NIPAM at 263.0 K; (b) simulation of NIPAM at 333.0 K; (c) simulation of IPOZ at 263.0 K; (d) simulation of IPOZ at 333.0 K.

a

b

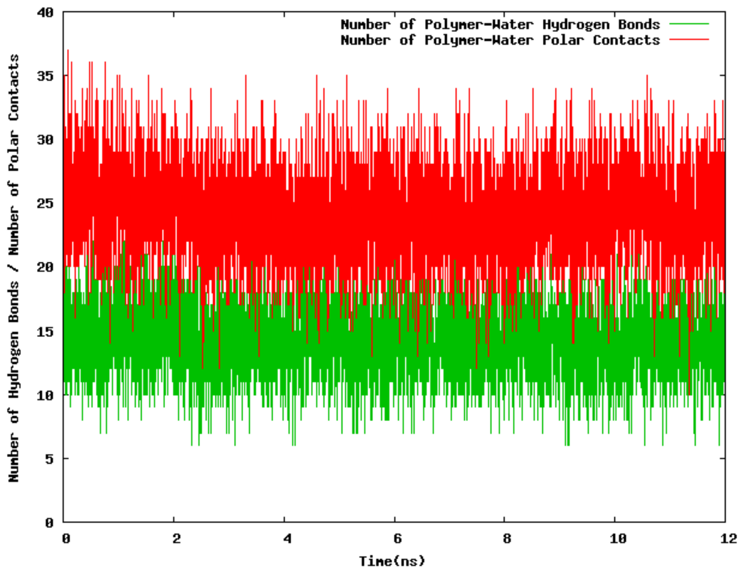

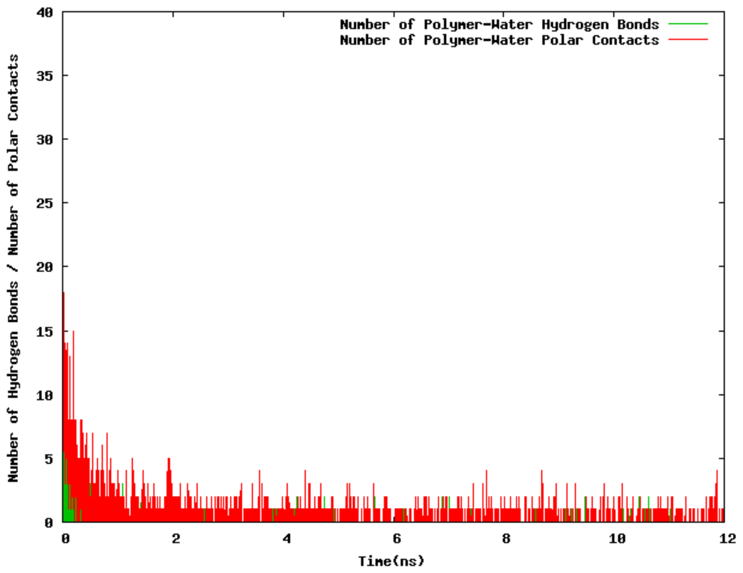

c

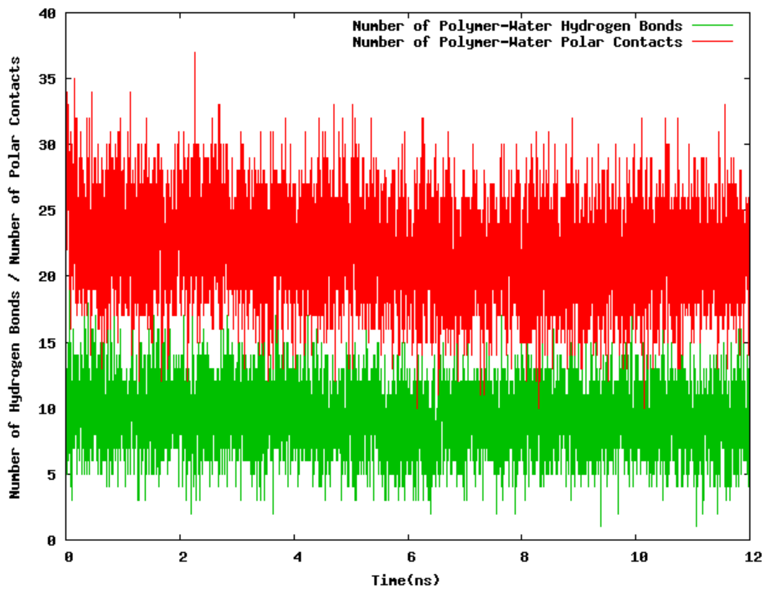

d

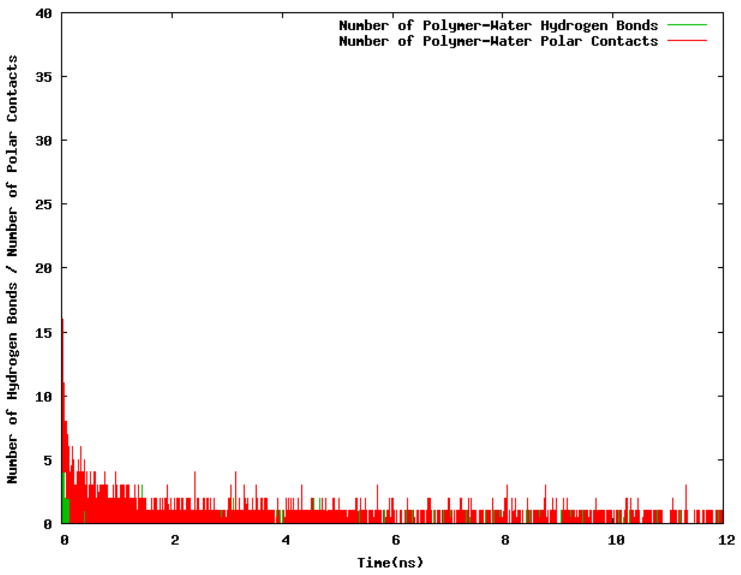

Figure S5. Variation with the simulation time of the number of hydrogen bonds and the contacts between polar atoms of the 12-mer NIPAM / IPOZ molecules and the solvent molecules, starting from initially extended conformations in a cubic box with 100 Å side: (a) simulation of NIPAM at 263.0 K; (b) simulation of NIPAM at 333.0 K; (c) simulation of IPOZ at 263.0 K; (d) simulation of IPOZ at 333.0 K.

Force Field Information

We applied GROMACS port of AMBER03 force field. It is a third-generation point-charge all-atom force field. AMBER03 force field is based on AMBER99 which is optimized for organic molecules. Partial charges are derived using the restrained electrostatic potential (RESP) approach. The functional form is given below. Though simple it gives accurate description of organic systems in terms of structures and non-bonded interactions (energies). As far as the lack of anharmonic and cross-terms is concerned, one can note that they are needed for strongly strained systems (not our case).

$$E = \sum_{bonds} K_b (b - b_{eq})^2 + \sum_{angles} K_\theta (\theta - \theta_{eq})^2 + \sum_{dihedrals} \frac{V_n}{2} \left[(1 + \cos(n\varphi - \gamma) \right] + \sum_{i<j} \left[\frac{A_{ij}}{R_{ij}^{12}} - \frac{B_{ij}}{R_{ij}^{6}} + \frac{q_i q_j}{\varepsilon R_{ij}} \right]$$

K_b force constant for the bond

K_θ force constant for the angle

b bond length

θ bond angle

b_{eq} equilibrium bond length

θ_{eq} equilibrium bond angle

φ dihedral angle

V_n force constant

γ phase angle

q_i partial charge (i-th atom)

ε dielectric constant

A_{ij} and B_{ij} Lennard–Jones terms
